# Albendazole-Schisandrin B Co-Therapy on *Angiostrongylus cantonensis*-Induced Meningoencephalitis in Mice

**DOI:** 10.3390/biom10071001

**Published:** 2020-07-05

**Authors:** Ho Yin Pekkle Lam, Ting-Ruei Liang, Shinn-Jong Jiang, Shih-Yi Peng

**Affiliations:** 1 Institute of Medical Sciences, Tzu Chi University, Hualien 97004, Taiwan; pekklelavabo@gmail.com; 2 Department of Biochemistry, School of Medicine, Tzu Chi University, Hualien 97004, Taiwan; 108330102@gms.tcu.edu.tw (T.-R.L.); sjjiang@mail.tcu.edu.tw (S.-J.J.)

**Keywords:** *Angiostrongylus*, meningoencephalitis, anthelmintic drug, albendazole, Schisandrin B, combination therapy

## Abstract

Currently, *Angiostrongylus cantonensis* infections are predominantly treated with albendazole. However, the use of albendazole can provoke certain neurological symptoms as a result of the immune response triggered by the dead worms. Therefore, treatment usually involves co-administration of corticosteroids to limit the inflammatory reaction. Corticosteroids play a useful role in suppressing inflammation in the brain; however, long-term usage or high dosage may make it problematic.Schisandrin B, an active ingredient from *Schisandra chinensis*, has been shown to have anti-inflammatory effects on the brain. This study aimed to investigate the effects and potential of schisandrin B in combination with albendazole to treat *Angiostrongylus*-induced meningoencephalitis. Here, we show that albendazole-schisandrin B co-treatment suppressed neuroinflammation in *Angiostrongylus*-infected mice and increased the survival of the mice. Accordingly, albendazole-schisandrin B co-treatment significantly inhibited inflammasome activation, pyroptosis, and apoptosis. The sensorimotor functions of the mice were also repaired after albendazole-schisandrin B treatment. Immune response was shown to shift from Th2 to Th1, which reduces inflammation and enhances immunity against *A. cantonensis*. Collectively, our study showed that albendazole-schisandrin B co-therapy may be used as an encouraging treatment for *Angiostrongylus*-induced meningoencephalitis.

## 1. Introduction

*Angiostrongylus cantonensis* is a parasitic nematode that is neurotropic and migrates to neural tissue after infection resulting in eosinophilic meningitis, encephalitis, and ocular angiostrongyliasis [1,2]. Because the course of eosinophilic meningitides in patients infected with *A. cantonensis* averages 1–4 weeks, the decision to start treatment should begin early [3]. Currently, *A. cantonensis* infections are treated predominantly with albendazole (Alb), which is an anthelmintic that acts by binding to β-tubulin of the parasite, inhibiting its glucose absorption and utilization. [4].Although sole treatment with Alb can effectively kill the worm, it cannot resolve the neuroinflammation induced by the worm carcasses [5]. Therefore, Alb treatment does not always achieve complete recovery of the brain. For this reason, treatment usually involves co-administration of corticosteroids to limit the inflammatory reaction [6,7,8]. Inclusion of corticosteroids, such as dexamethasone, has been used for a long time in the clinic and has played a useful role in suppressing inflammation in the brain.However, long-term usage or high dosage may be problematic by causing several adverse effects, such as immunodepression, adrenal suppression, and gastroesophageal reflux [3,9]. In order to increase the survival rate and quality of treatment, it may be helpful to replace the use of steroids with other anti-neuroinflammatory agents.

Schisandrin B (Sch B) is a bioactive ingredient isolated from the plant, *Schisandra chinensis* (also known as the five-flavor berry or Wu Wei Zi). Several lines of evidence have shown that Sch B has anti-oxidative, anti-inflammatory, and anti-tumor activities [10,11,12]. Recent studies have shown that Sch B can be used as a treatment for bacterial or lipopolysaccharide-induced neuroinflammation [13,14,15]. Moreover, many studies have been conducted on the protective effects of Sch B on central nervous system (CNS) diseases [14,15,16,17,18]. However, the effect of Sch B on *Angiostrongylus*-induced meningoencephalitis is not clear.

More than two immune responses have been known to regulate inflammation in the host response to infection, including inflammasome activation and cytokine production. Inflammasome activation plays a role in inflammation by releasing inflammatory cytokines, such as interleukin (IL)-1βand IL-18 [19]. A recent study has shown that *A. cantonensis* infection induces activation of NACHT, LRR, and PYD domains-containing protein 1B (NLRP1B) and NLR family CARD domain-containing protein 4 (NLRC4) inflammasomes in a mouse model [20]. In addition, *A. cantonensis* infection induces pyroptosis via ASC-independent gasdermin D (GSDMD)cleavage [20].In contrast, *A. cantonensis* infection upregulates Th2 cytokines (IL-4 and IL-10) and downregulates Th1 (IL-2 and IFN-γ) in both human and mouse models [21,22]. Both inflammation and cytokines are involved in the process of inflammation. Therefore, we aimed to examine the effect of Sch B on inflammasome activation and cytokine production. In this study, infected mice were treated with Sch B and Alb to investigate the effects on *Angiostrongylus*-induced meningoencephalitis.

## 2. Methods

### 2.1. Parasites and Animals

The parasite life cycle was maintained as previously described [20]. Third-stage larvae (L3) of *A. cantonensis* was isolated by the Baermann apparatus from the giant African snail, *Achatina fulica* in Taipei. L3 were used to infect Sprague-Dawley (SD) rats, which were used as the final host, to maintain the parasite life cycle. First-stage larvae (L1) were isolated from the feces of infected rats. Fresh-water snails, *Biomphalaria glabrata*, were used as intermediate host and were infected with the acquired L1 for 21 days. On day 21 post-infection, the snails were killed and their tissues were digested with 0.6% pepsin (pH 2). L3 were isolated from the snails using the Baermann apparatus. The isolated L3 were used to infectBagg Albino (BALB/c) mice for the experiment or infect SD rats to maintain the life cycle of the parasite. Mice were used for the experiment because both the mice and human act as non-permissive host for *A. cantonensis* and induce similar pathological changes. Permissive rat can survive *A. cantonensis* infection and cause less severe CNS damage [23,24]. SD rats and BALB/c mice used in this study were purchased from the National Laboratory Animal Center, Taipei. All protocols involving animals were approved by the Institutional Animal Care and Use Committees (IACUC) of Tzu Chi University (No.107086).

### 2.2. Animal Treatment

**Figure 1:**
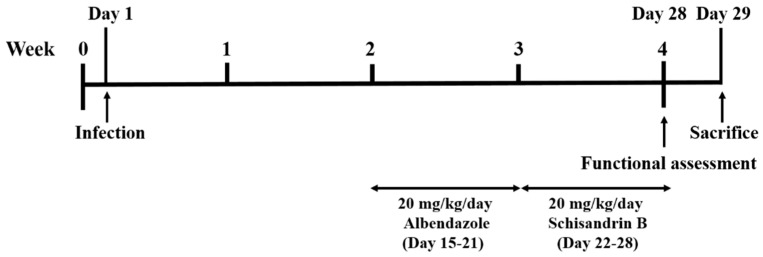
Experimental design scheme.

Mice were equally and randomly divided into five groups—one control, one infection, and three treatment groups, in which each group contained at least 10 mice. Mice in the infection and treatment groups were infected with 25 L3 by oral gavage, and the control group was treated with normal saline. For the Alb treatment group, mice were treated with 20 mg/kg/day Alb (A4673, Sigma Aldrich, St. Louis, MO, USA) for 7 consecutive days from week 2 post-infection (days 15–21). For the Sch B treatment group, mice were treated with 20 mg/kg/day Sch B (61281-37-6, Chengdu Alfa Biotechnology, Chengdu, China) for 7 consecutive days from week 3 post-infection (days 22–28) [25]. For the co-treatment group, mice were treated with 20 mg/kg/day Alb and 20 mg/kg/day Sch B for 7 consecutive days from week 2 and week 3 post-infection, respectively. All treatments were administered orally, and all groups were subjected to functional tests at week 4 post-infection. One day later (day 29), the mice were sacrificed. The experimental design scheme is shown in Figure 1. Upon mouse dissection, brain specimens were obtained for further studies.

### 2.3. RNA Isolation, cDNA Synthesis, and Real-Time Quantitative PCR (qPCR)

Total RNA was isolated from brain tissues using TRIzolreagent (Thermo Scientific, Rockford, IL, USA),according to the manufacturer’s instructions. RNA (5 μg) was used for cDNA synthesis in a 20 μL reaction mixture using the RevertAid First Strand cDNA Synthesis Kit (Fermentas International Inc., Burlington, ON, Canada). Reverse transcription PCR (RT-PCR) was performed at 50 °C for 1 h, followed byextension for 15 min at 70 °C. The synthesized cDNA was stored at −20 °C for qPCR experiments. qPCR was performed with the LabStar SYBR qPCR Kit(Bioline, London, UK) using the Roche LightCycler 480 System. The primers used in this study are shown in Table 1. Relative gene expression was calculated using the ΔΔCt method and gene expression levels were normalized to the internal control β-actin.

### 2.4. Protein Extractionand Western Blot Analysis

The brain tissues of the mice were washed with PBS and 200 μL RIPA buffer (Thermo Scientific). The cell lysates were incubated for 30 min at 4 °C and centrifuged at 8100× *g* for 10 min at 4 °C. Extracted proteins were separated on 10% or 12% SDS-PAGE gels and the gels were transferred to a PVDF membrane (EMD Millipore, Burlington, MA, USA). Membranes were blocked with 5% nonfat milk prior to overnight incubation at 4 °C with agitation with the following primary antibodies: α-tubulin (GTX628802, GeneTex, Irvine, CA, USA), NLRP1B (SC-390133, Santa Cruz Biotechnology, Dallas, TX, USA), NLRC4 (06-1125, EMD Millipore), caspase-1 (22915-1-AP, Proteintech, Rosemont, IL, USA), IL-18 (10663-1-AP, Proteintech, Rosemont, IL, USA), IL-1β (16806-1-AP, Proteintech, Rosemont, IL, USA),Caspase-3 (GTX110543,GeneTex, Irvine, CA, USA), Caspase-8 (GTX110723, GeneTex, Irvine, CA, USA), Caspase-9 (GTX112888, GeneTex, Irvine, CA, USA), and BCL-2 (GTX100064, GeneTex, Irvine, CA, USA). The membranes were incubated with the HRP-conjugatedmouse anti-IgG (EMD Millipore) or HRP-conjugated rabbit anti-IgG (EMD Millipore) secondary antibodies for 1 h, following which the membranes were developed using ECL detection reagent (EMD Millipore). Relative protein levels were quantified using Image J (Version 1.46, National Institute of Health, Bethesda, MD, USA), and protein densitometry was expressed relative to that of α-tubulin.

### 2.5. Hematoxylin and Eosin Staining

Mouse brains were harvest and immediately fixed with 10% formalin for 48 h. After fixation, tissues were dehydrated in a series of graduated changes of alcohols: 70% (2 × 30 min), 95% (2 × 30 min), 100% (2 × 30 min). The procedures were followed by immersion in xylene (2 × 30 min) and paraffin bath (30 min). Tissues were then put in an embedding cassette with melted paraffin. The cassette was transferred to an ice tray for 30 min to allow complete hardening of the paraffin. The paraffin-embedded tissues were then sectioned into 5–10 µm thin slices using a microtome and mounted onto slides. The slides were dry overnight at room temperature. Before proceeding with the staining protocol, the slides were deparaffinized and washed with Sub-X (Leica Biosystems, Richmond, IL, USA) and 100%, 95%, 75%, and 50% ethanol. The rehydrated sections were stained as follows: hematoxylin solution (Merck, Darmstadt, Germany) (3 min), tap water (1 min), 70% ethanol with 1% HCl (5 s), tap water (1 min), eosin solution (3 min), 95% ethanol (3 × 5 min), 100% ethanol (2 × 5 min), and Sub-X (3 × 15 min).

### 2.6. Preparation of Mouse Brain Cells

Mouse brains were enzymatically dissociated using 0.25% trypsin-EDTA (GeneDireX, Taoyuan, Taiwan) diluted in Dulbecco’s Modified Eagle Medium (DMEM) (Gibco, Waltham, MA, USA) and incubated at 37 °C for 10 min. Gentle pipetting was perform to break up tissues clumps. The process was repeated until the tissues were completely lysed. The cells were filtered through a 40-μm strainer and centrifuged for 5 min at 1000× *g*. The precipitate was resuspended in DMEM [20].

### 2.7. Annexin V-FITC/Propidium Iodide (PI) Double Staining by Flow Cytometry

Apoptotic cell death was measured using the Alexa Fluor Annexin V/Dead Cell Apoptosis kit (Molecular Probes Inc., Eugene, OR, USA) following the manufacturer’s protocol. The brain cells were washed and resuspended in ice-cold binding buffer, and stained with 5 μL Annexin V-FITC and PI in the dark for 15 min. Thereafter, 400 μL binding buffer was added to the mixture, and the cells were analyzed using a Gallios™ 10-channel flow cytometer (Beckman Coulter, Brea, CA, USA). A minimum of 1 × 10^4^ events in the “cell” area of the FSC versus SSC dot plots were collected per sample.

### 2.8. Collection of Cerebrospinal Fluid (CSF)-Like Fluid

The cranial cavity and cerebral ventricles of the mouse brain were rinsed with 1 mL PBS. The washing solution was collected and centrifuged at 600× *g* for 10 min. The supernatant was collected and centrifuged at 10,000× *g* for 30 min. The supernatant acted as the CSF, so called ‘CSF-like fluid’ [26], which was stored at −20 ℃ until use.

### 2.9. Measurement of Lactate Dehydrogenase (LDH)

LDH levels were used as a parameter of cell pyroptosis. Whole blood was obtained by cardiac puncture and was centrifuged at 600× *g* for 10 min to obtain the serum. Serum was then analyzed for LDH using a Hitachi 7080 Chemistry Analyzer (Hitachi Ltd., Tokyo, Japan).

### 2.10. Hindlimb Clasping Test

Mice were suspended by their tail for 10 s. Suspended mice were observed, and a score was assigned based on the following criteria. A score of 0 was given if the mice showed normal escape extension and both hindlimbs were splayed outward away from the abdomen. A score of 1 was given if the hindlimb was retracted toward the abdomen but not touching the abdomen. A score of 2 was given if the hindlimb was retracted and touched the abdomen while the hindlimb was not clasped. A score of 3 was given if the mice showed immobility and both hindlimbs were clasped and touched the abdomen [27,28].

### 2.11. Beam Walk Test

A beam, 70 cm long and 1 cm in diameter, was placed horizontally 50 cm above the platform. The mice were trained for 2 days before the test. Mice were habituated in the goal box for 5 min and placed at a distance of 20 cm from the goal box on the beam. The mouse was trained to walk on the beam and reach the goal box without difficulty. On the day of the test, the mice were placed at the start point of the beam (70 cm from the goal box). The time for the mouse to cross the beam and reach the goal box was recorded with a 120 s cutoff time. The number of times the mice slipped during the walk was also recorded [27]. Each mouse was tested for three trials, and the average was calculated.

### 2.12. Vertical Pole Test

A 50 cm plastic pole was placed vertically in a large mouse cage with bedding. Mice were placed at the top of the pole and the time for it to turn around and climb down was recorded with a 120 s cutoff time. If the mice fell from the pole, time was recorded as 120 s [27]. Each mouse was tested for three trials, and the average was calculated.

### 2.13. Hot Plate Test

Mice were placed on a hot plate at a temperature of 55 °C, and the time for the first paw reaction (jumping or paw licking) was recorded. A cutoff time of 15 s was considered to avoid damage to the paws. The test was repeated at 15 min intervals. Each mouse was tested for three trials, and the average was calculated.

### 2.14. Cytokine ELISA

Concentrations of IL-4, IL-10, IL-2, and IFN-γ in the sera and CSF were measured using a standard sandwich ELISA kit (Thermo Fisher Scientific, Waltham, MA, USA), according to the manufacturer’s instructions. Briefly, 96-well ELISA plates were coated overnight at 4 °C with 100 μL capture antibody. The next day, the capture antibody was discarded, wells were washed, and 200 μL ELISA/ELISASPOT diluent was added. After 1 h incubation, the wells were washed, 100 μL samples, and standards were added and incubated for 2 h. Then, plates were washed and 100 μL detection antibody was added and incubated for 1 h. The wells were then washed, and 100 μL Avidin-HRP enzyme was added and incubated for 30 min. After washing, 100 μL 3,3’,5,5’-Tetramethylbenzidine (TMB) substrate was added and incubated for 15 min. Sulfuric acid (10%) was applied to stop the reaction, and the optical density was determined at 450 nm. The sensitivity of the kit for IL-4, IL-10, IL-2, and IFN-γ was 4 pg/mL, 30 pg/mL, 2 pg/mL, and 15 pg/mL, respectively.

### 2.15. Statistical Analysis

All experimental data were analyzed using GraphPad Prism 6.01 (GraphPad Software, San Diego, CA, USA). Data are represented as the mean ± S.D. One-way analysis of variance (ANOVA) was used, followed by a Tukey’s post-hoc test to determine differences between groups. A *p*-value < 0.05 indicates a significant difference.

## 3. Results

### 3.1. Albendazole-Sch B Co-Therapy Ameliorates Brain Damage in A. Cantonensis-Infected Mice

**Figure 2:**
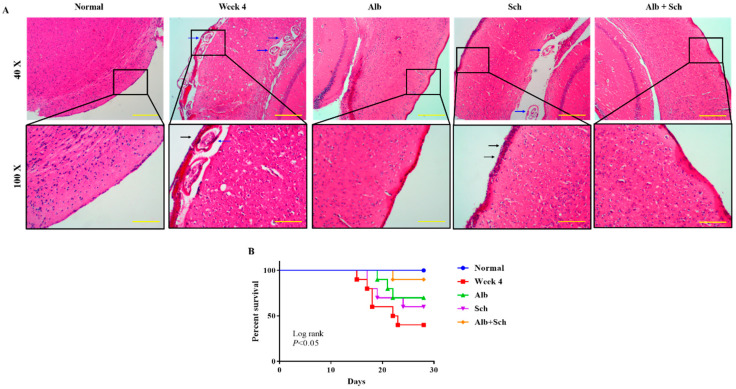
Alb-Sch B co-treatment ameliorates brain damage and increases the survival rate in *A. cantonensis*-infected mice. (**A**) Representative images showing hematoxylin and eosin (HE) staining of brain sections of normal (uninfected and untreated) mice, infected mice, infected mice with Alb treatment, infected mice with Sch B treatment, and infected mice with Alb-Sch B co-treatment. Images are shown at 40× (Scale bar, 500 μm) and 100× (Scale bar, 200 μm) magnification. The blue arrows show the trapped larvae. Black arrows show infiltration of inflammatory cells. (**B**) Mice from each group were monitored daily for survival until the day of sacrifice (day 29). Statistical analysis was performed using Kaplan–Meier estimate with the Mantel-Cox log-rank test, *p* < 0.05.

To investigate the protective effect of Sch B on the brain of *Angiostrongylus*-infected mice, hematoxylin and eosin staining was performed. When mice were infected with *Angiostrongylus* for 4 weeks, a thickened meninx was observed, which was expanded by large amounts of infiltrated inflammatory cells. Trapped worms were also observed in the meninges and cortex. When mice were treated with Alb alone, a still thickened but reduced severity of meningitis was observed. However, when mice were treated with Sch B alone, it failed to diminish the inflammatory responses. In addition, trapped worms could still be found in the cortex area. When mice were co-treated with Alb and Sch B, meninges showed much improvement with less inflammatory cells compared to those treated with Alb alone (Figure 2A). As inflammation was reduced in the brain after co-treatment with Alb and Sch B, we also observed an increased survival of these mice (Figure 2B).

### 3.2. Albendazole-Sch B Co-Therapy Inhibits Inflammasome Activation and Pyroptosis in A. Cantonensis-Infected Mice

**Figure 3:**
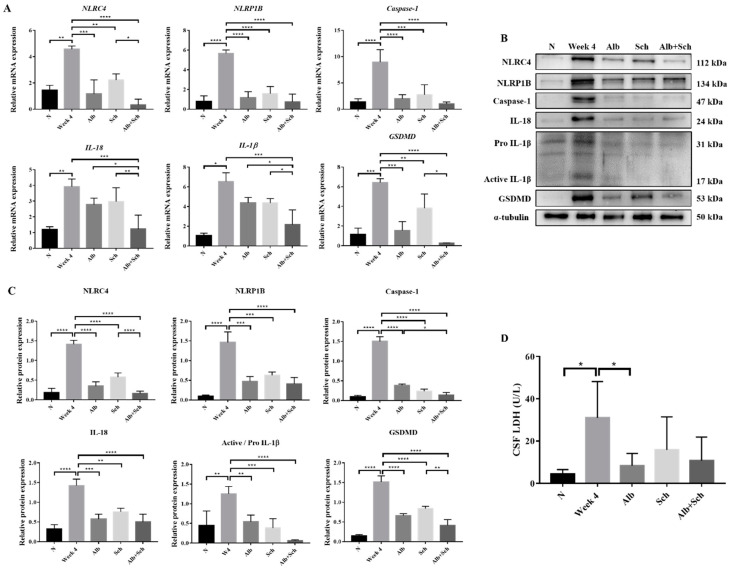
Alb-Sch B co-therapy inhibits inflammasome activation and pyroptosis in *A. cantonensis*-infected mice. (**A**) RNA transcription levels of inflammasome components, including NLRC4, NLRP1B, Caspase-1, IL-18, IL-1β, and gasdermin D (GSDMD),were measured by qPCR. Data are presented as the mean ± SD (n = 5). (**B**) Representative western blot images reflecting protein levels ofinflammasome components. (**C**) Bar graphs showing protein expression levels relative to that of α-tubulin. Results are presented as the mean ± SD (n = 3). (**D**) Lactate dehydrogenase (LDH)levels measured in the cerebrospinal fluid (CSF), shown as the mean ± SD (n = 5). * *p*-value < 0.05, ** *p*-value < 0.01, *** *p*-value < 0.001, and **** *p*-value < 0.0001.

Inflammasome activation plays a major role in producing inflammatory cytokines, such as IL-1βand IL-18, and induces host defenses against pathogens [19]. To examine the mechanism of Alb-Sch B co-treatment on resolving *Angiostrongylus*-induced meningoencephalitis, we assessed the mRNA and protein expression levels of the inflammasome components. A previous study has shown that *A. cantonensis* induces NLRC4 and NLRP1B activation and pyroptosis in the mouse brain [20]. Consistent with this study, our data revealed increased expression levels of inflammasome activators (NLRC4 and NLRP1B) and inflammasome effectors (Caspase-1, IL-1β, and IL-18) in *A. cantonensis*-infected mice. In contrast, Alb and Sch B decreased the levels of inflammasomes (Figure 3A–C). Interestingly, Sch B treatment alone also reduced inflammasome activation in the infected mice, despite having little to no observable effect on the histological sections and mice survival (Figure 2A,B). Similarly, Alb-Sch B co-treatment further reduced the level of inflammasomes compared to treatment with Alb or Sch B alone. Pyroptosis can be induced by caspase-1-mediated cleavage of GSDMD [29]. Once GSDMD is cleaved, it forms pores on the cell membrane and releases intracellular content [29]. As expression of GSDMD in the brain increased with *A. cantonensis* infection and reduced after treatment (Figure 3A–C), we measured the level of LDH in the CSF as an indicator of pyroptosis presence [30]. Similarly, LDH levels in the CSF increased significantly during infection and decreased after treatment with Alb and Sch B (Figure 3D). Collectively, these data demonstrate that Alb-Sch B treatment can inhibit inflammasome activation and pyroptosis.

### 3.3. Albendazole-Sch B Co-Therapy Alleviates Apoptosis in A. Cantonensis-Infected Mice

**Figure 4:**
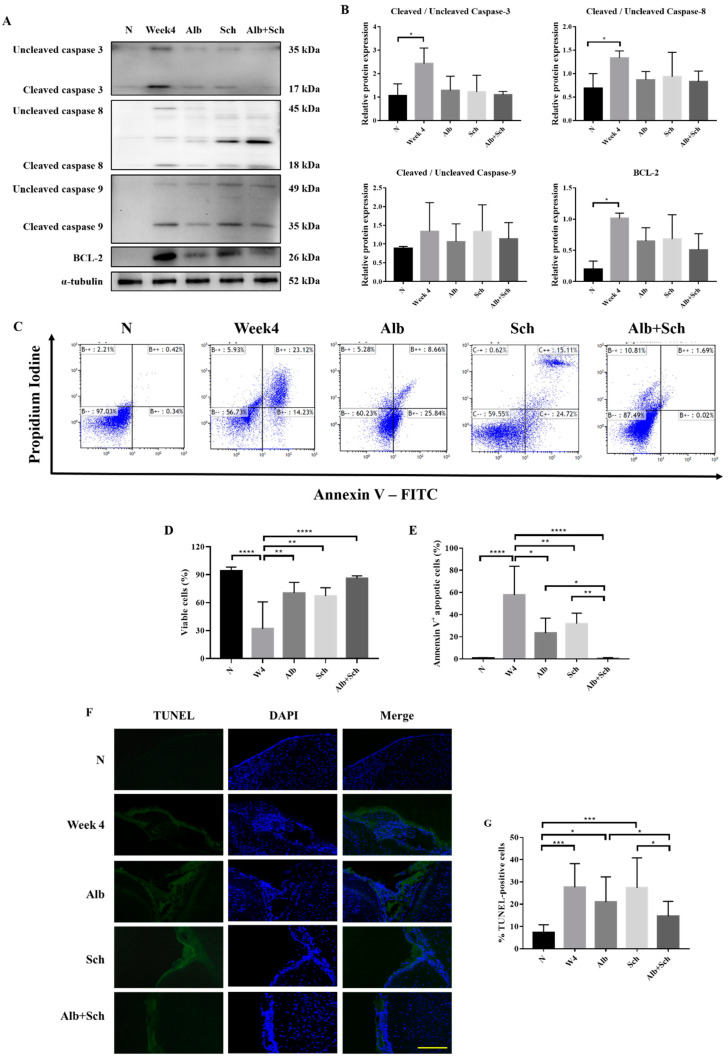
Alb-Sch B co-therapy inhibits apoptosis in *A. cantonensis*-infected mice. (**A**) Representative western blot images reflecting protein levels of Caspase-3, Caspase-8, Caspase-9, and BCL-2. (**B**) Bar graphs showing protein expression levels relative to that of α-tubulin. Results are presented as the mean ± SD (n = 3). (**C**) Representative plots from one set of Annexin V-FITC and PI double staining, measured by flow cytometry. Viable cells are shown in the bottom left quadrant (double negative); early apoptotic cells are shown in the bottom right quadrant (Annexin V positive and PI negative); late apoptotic cells are shown in the upper right quadrant (double positive). (**D**) Percentages of viable cells, shown as the mean ± SD (n = 5). (**E**) Percentages of apoptotic cells (including both early apoptotic and late apoptotic cells) are shown as the mean ± SD (n = 5). (**F**) Representative microphotographs of TUNEL staining. Scale bar, 200 μm. (**G**)Percentages of TUNEL-positivecells, quantitated by counting ratio of TUNEL/DAPI in 5 random microscopic fields, shown as mean±SD (n = 3). * *p*-value < 0.05, ** *p*-value < 0.01, *** *p*-value < 0.001, and **** *p*-value < 0.0001.

Apoptosis is mainly achieved by caspase activation. We therefore investigated the main apoptotic caspases involved, including caspase-3, caspase-8, and caspase-9, and the antiapoptotic protein BCL-2. Compared with the control group, the protein expression of apoptotic caspase apparently increased during *A. cantonensis* infection, whereas treatment with Alb-Sch B reversed these effects (Figure 4A,B). In accordance with the expected change in apoptotic caspase, we observed a similar trend in BCL-2 (Figure 4A,B). We suggest that the increase in BCL-2 is the compensation mechanism for the increase in apoptotic caspases. To complement the western blot experiments, the mouse brain cells were stained with Annexin V and PI (Figure 4C). It can be seen clearly that the brain cells experience severe apoptosis during infection, whereas treatment with Alb-Sch B resolves the damage (Figure 4D,E). In addition, terminal deoxynucleotidyl transferase-mediated dUTP nick-end labeling (TUNEL) staining showed increased TUNEL-positive cells in *A. cantonensis*-infected mice. Treatment with Alb-Sch B reduced the number of TUNEL-positive cells (Figure 4F,G). Together, these data suggest that Alb-Sch B co-treatment can alleviate apoptosis in *A. cantonensis*-infected mice.

### 3.4. Albendazole-Sch B Co-Therapy Repairs Sensorimotor Function in A. Cantonensis-Infected Mice

**Figure 5:**
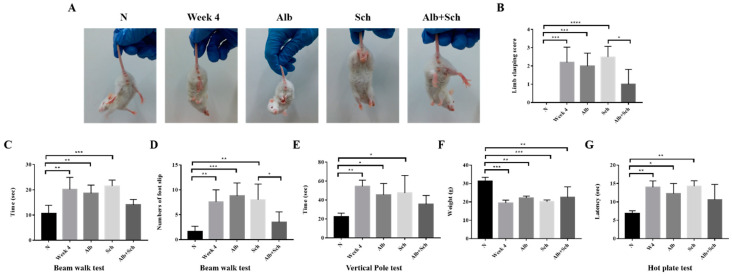
Alb-Sch B co-therapy repairs sensorimotor function in *A. cantonensis*-infected mice. (**A**) Representative image of mice in the hindlimb clasping test. (**B**) Quantitative analysis of hindlimb clasping scores. The infection group and mice treated solely with Alb or Sch B showed higher scores than normal mice. Alb-Sch co-treated mice showed a lower score. (**C**,**D**) Results of the beam-walk test. The infection group and mice treated solely with Alb or Sch B took longer to finish the test and had a greater number of foot slips. Alb-Sch co-treated mice showed better performance on the test. (**E**) Vertical pole test results. Infected mice and single drug-treated mice took longer to finish the test, whereas Alb-Sch B co-treated mice used less time to finish the test. (**F**) Body weight of mice. (**G**) Hot plate test results. A longer time was needed for the infected and single drug-treated mice to sense pain stimuli. After Alb-Sch B treatment, mice use less time to respond to pain. All results are presented as the mean ± SD (n = 5–10). * *p*-value < 0.05, ** *p*-value < 0.01, ***p-value < 0.001, and **** *p*-value < 0.0001.

As Alb-Sch B co-treatment reduced neuroinflammation and apoptosis in *Angiostrongylus*-infected mice, we tested the sensorimotor function of the mice. The hindlimb clasping test is used as an indicator of motor function. Uninfected mice showed a normal escape extension reflex and yielded lower scores. However, upon infection, the mice showed a higher score, indicating motor dysfunction. Mice treated solely with Alb or Sch B showed similar scores to those of the infection group. After Alb-Sch B co-treatment, the clasping score improved (Figure 5A,B). The beam walk test was used to investigate the motor coordination of the mouse. A longer time was used by the infected and single drug-treated mice to cross the beam than the uninfected mice; moreover, a greater number of slips was observed in these mice. Similarly, Alb-Sch B co-treated mice showed improvement in this test (Figure 5C,D). Results of the vertical pole test show that infected and single drug-treated mice took longer to turn around and climb down the pole than normal mice. In mice co-treated with Alb-Sch B, a better performance was observed (Figure 5E). We also measured the body weight of each mouse, as motor function is dependent of both the central nervous system and skeletal muscles [31]. The mice became emaciated and a decreased body weight was observed after mice were infected with *A. cantonensis*, which may suggest a decrease in muscle strength. After Alb-Sch B co-treatment, although the mice only showed a non-statistically significant increase in body weight (Figure 5F), they were generally heavier, with smoother furs, and a healthier appearance. The hot plate test was used to assess the mice’s ability to sense pain stimuli. As shown in the results, the mice were not able to respond to a pain stimulus after *A. cantonensis* infection. Co-treatment with Alb-Sch Bcan slightly but not significantly improve the response of the mice (Figure 5G). Collectively, these data indicate that Alb-Sch B treatment can repair the sensorimotor function of mice with *A. cantonensis* infection.

### 3.5. Alb-Sch B Co-Therapy Regulates Th1 and Th2 Immune Responses in A. Cantonensis-Infected Mice

In both *Angiostrongylus*-infected human and BALB/c mice, Th2 cytokines (IL-4 and IL-10) were dominant, whereas Th1 cytokines (IL-2 and IFN-γ) remained at a low level [21,22]. To better understand how Alb and Sch B modulate the host response to *A. cantonensis* infection, we examined both local and systemic cytokine responses.

**Figure 6:**
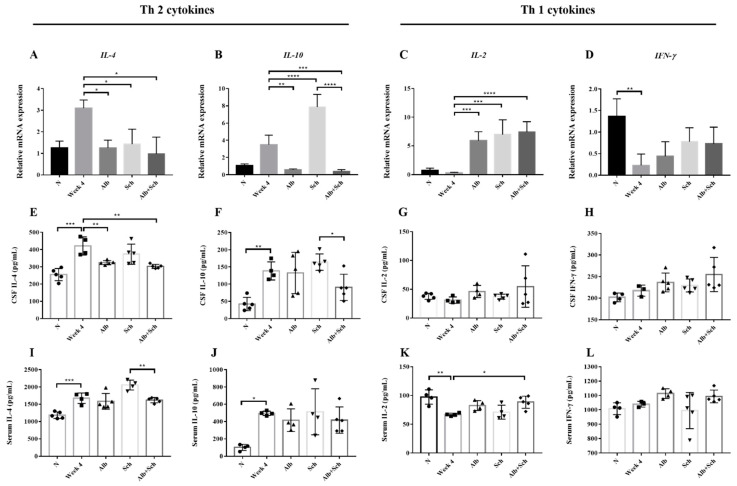
Alb-Sch B co-therapy regulates Th1 and Th2 immune responses in *A. cantonensis*-infected mice. (**A**–**D**) Relative mRNA expression levels of IL-4, IL-10, IL-2, and IFN-γ. (**E**–**H**) Concentrations of IL-4, IL-10, IL-2, and IFN-γ in the CSF, measured by ELISA. (**I**–**L**) Concentrations of IL-4, IL-10, IL-2, and IFN-γ in the serum, measured by ELISA. IL-4 and IL-10 are Th 2 cytokines; IL-2 and IFN-γ are Th1 cytokines. All results are presented as the mean ± SD (n = 3–5). * *p*-value < 0.05, ** *p*-value < 0.01, *** *p*-value < 0.001, and **** *p*-value < 0.0001.

IL-4, IL-10, IL-2, and IFN-γ levels were measured by qPCR and ELISA. Both brain mRNA levels and cytokine concentrations of IL-4 and IL-10 were shown to be upregulated after infection with *A. cantonensis* (Figure 6A,B), whereas those of IL-2 and IFN-γdecreased (Figure 6C,D). These data corroborated previous data [21,22] and indicatethat *A. cantonensis* infection stimulates Th2 responses. When mice were co-treated with Alb and Sch B, it can be observed that the *IL-4* and *IL-10* mRNA levels were decreased (Figure 6A,B), indicating that co-treatment with Alb and Sch B ameliorated the Th2 immune response caused by *A. cantonensis*. The cytokine concentrations with respect to IL-4 and IL-10 were also measured in the serum and CSF. Similarly, the concentrations of both IL-4 and IL-10 increased during infection and decreased after co-treatment with Alb and Sch B (Figure 6E,F,I,J). In contrast, mRNA levels and cytokine concentrations of IL-2 and IFN-γ decreased after infection, but increased after treatment (Figure 6C,D,G,H,K,L). Together, these results suggest that Sch B, when co-treated with Alb, can shift the Th2 immune response to the Th1 immune response.

## 4. Discussion

In the present study, we first demonstrated that neuroinflammation was significantly reduced with Alb-Sch B co-treatment. In addition, reduced inflammation led to an increased survival rate. The suppression of inflammasome components and pyroptosis may explain the mechanism of the neuroprotective effects of Sch B. Our study results are similar to those of Guo et al. [13]. In their study, they found that Sch B effectively suppresses inflammasome activation and pyroptosis and therefore inhibits inflammation [13]. Recent studies also suggested that Sch B is effective in reducing neuroinflammation in various neuro-disease models, such as cerebral ischemia in rats [18], lipopolysaccharide-induced microglia [15], and 6-OHDA-induced Parkinson’s disease in mice [17].

In addition, decreased apoptosis was found after co-treatment with Alb and Sch B, whereas the decrease was not significant when treated with Alb or Sch B alone. Previous studies have identified the role of Sch B in inducing cell apoptosis through caspase activation and BCL-2 inhibition in different cancer cells [32,33,34]. In the current study, we found that Sch B inhibits caspase activation. This was seen when *A. cantonensis*-infected mice were treated with Sch B alone or in combination with Alb (Figure 4A,B). This inhibition of apoptotic caspase is also seen in the treatment of neurological disorders, such as Alzheimer’s disease [16,35] and amyloid β-induced neurotoxicity [36]. Therefore, we propose that Sch B has a neuroprotective effect in an *A. cantonensis*-infected mouse model by downregulating inflammation and apoptosis.

In addition to inflammasome activation and apoptotic caspase activation, hypoxia is also found associated with neuroinflammation by activating microglia [37]. Multiple lines of evidences have pointed to the bidirectional relationship between hypoxia and inflammation. Not only hypoxia induces tissue inflammation, inflamed tissues can also cause hypoxia [38]. Hypoxia-inducible factor-1 (HIF-1) is a transcription factor that control hypoxia-activating genes, such as erythropoietin (EPO) [39], and can be positively linked with pathogen infection [40]. Regulated in development and DNA damage responses 1 (REDD1), a HIF-1-response gene, is strongly induced during hypoxia and is positively related with inflammation [41,42,43]. Previous study indicated a role for REDD1 in controlling autophagy and mitochondrial function [44], which may affect mice behaviors [45,46]. It is perhaps unsurprising that *A. cantonensis*-induced brain cell inflammation also induce hypoxia. However, the role of hypoxia in *A. cantonensis* infection remains unclear and further studies are needed to confirm our hypothesis.

To further clarify the neuroprotective effect of Alb-Sch B treatment, we performed different sensorimotor tests on the mice. The data revealed that *A. cantonensis* infection impairs motor control of the mice, which is largely owing to damage to the central nervous system. The sole use of Alb or Sch B cannot improve the performance of the motor tests, indicating that it cannot effectively cure the host with *A. cantonensis* infection. However, using Alb and Sch B together can yield improvement in motor function, which also increases the survival rate of these mice. Although Alb can kill the worm, it cannot ameliorate the immune response caused by the trapped dead worms [5], and eventually leads to neuroinflammation and brain cell apoptosis. Sch B, in contrast, cannot kill the worm but can reduce the severity of inflammation. Both these drugs, when used alone, cannot yield an improvement to mice. However, when used together, a significantly better improvement in neuro-related functions was observed.

We hypothesized that the improvement in inflammation and neurological function may be due to immunoregulation. We therefore tested the cytokine levels in the mice. Our results showed increased Th2 (IL-4 and IL-10) responses after *A. cantonensis* infection, which is similar that reported by a previous study [22]. IL-4 is an important cytokine that helps in higher brain functions, such as memory and learning [47]; IL-1β, which we also found increased during infection, has been suggested to exert a negative effect on cognitive behavior, characterized by sickness, depression, and stress [48,49]. We therefore suggest that the increase in IL-4 may compensate for the increase in IL-1β, which affected the neuro-related behavior of the mice. In addition to altering the neurological behavior, IL-4 can also enhance immunity against *A. cantonensis* infection by activating B cells and inducing a class switch of IgE, which helps in defending parasite infection [50,51]. Several studies have indicated that IL-4 can influence the progression of neurological diseases, such as Alzheimer’s disease, multiple sclerosis, and glioblastoma multiforme [52]. IL-4 was then found to decrease after Alb-Sch B co-treatment, which is consistent with the improved neurological behavior and decreased IL-1β. IL-10, in contrast, is an anti-inflammatory cytokine [53,54]. IL-10 exerts its effects by inhibiting MHC class II expression on antigen-presenting cells, thereby blocking the production of proinflammatory cytokines [55]. IL-10 has also been shown to be produced and act as an anti-inflammatory cytokine during parasitic infection with *Plasmodium* spp. [56], *Toxoplasma gondii* [57], and *Trypanosoma cruzi* [58,59]. IL-10 deficiency was shown to be involved in fibrosis caused by *Schistosoma mansoni* [60]. Therefore, we suggest that the increase in IL-10 is to strike a balance between anti-inflammation and inflammation during *A. cantonensis* infection. After co-treatment with Alb and Sch B, IL-10 levels were found to be decreased compared with those in the infection group. This result corroborates the theory that Alb-Sch B co-treatment can better improve neurological damage caused by *A. cantonensis* than a single drug treatment. Treatment with Sch B alone unexpectedly resulted in higher levels of IL-10 (Figure 6B,F,J), which is inconsistent with other reports that Sch B lowered the level of IL-10 [61]. However, combination therapy with Alb yields an expected low level of IL-10. The mechanism underlying this increase in IL-10 needs further study.

The Th1 (IL-2 and IFN-γ) response was initially found to be low during infection with *A. cantonensis* but increased after Alb-Sch B co-treatment. Infection with *A. cantonensis* has been confirmed to lower IL-2 and IFN-γ production in both humans and mice [21,22], which corroborates our results. It is known that IL-2 is necessary for the generation of Th1 and Th2 cells and to provide immune functions [62]. The absence of IL-2 has been confirmed to cause inflammationand autoimmune responses in multiple organs [63,64,65]. Therefore, we propose that the increase in IL-2 levels after treatment is to enhance host immunity against *A. cantonensis*. IFN-γ, although well known for its pro-inflammatory properties, has also been shown to have anti-inflammatory effects [66]. In addition, several studies have indicated a neuroprotective role for IFN-γ [67,68,69,70]. Notably, the production of IFN-γ-induced inflammation can also be blocked by IL-10 [55,71], which is consistent with the changes in neurobehavior, histology, and IL-10 levels during *A. cantonensis* infection and treatment with Alb and Sch B.

Our current findings support the theory that the combined use of Alb and Sch B yielded better results than Alb monotherapy. The improvement was attributed to reduced brain cell inflammation and apoptosis. Increased survival was also correlated with better neurological performance. We hypothesize that co-therapy with Alb-Sch B regulates immune function in mice by shifting Th2 response to that of Th1 response, thereby inhibiting inflammation and exerting neuroprotection. However, further investigation is needed to confirm the role of cytokines in the recovery of *A. cantonensis* infection. Noticeably, our results found thatthe anti-inflammatory effect of Sch B was not significant when *A. cantonensis* is still alive, possibly because the worm excretes some inflammatory mediators which Sch B cannot effectively inhibit. Thus, Sch B exert anti-inflammatory effects only after *A. cantonensis* is killed by Alb. Collectively, the current findings not only support the theory that Sch B may be used in combination with Alb to treat *A. cantonensis* infection but also provide an understanding of how Sch B modulates immune function and inflammation.

## Figures and Tables

**Table 1:** Primer pairs of candidate genes used in real time quantitative PCR.

Gene Name	Primer Pairs	Melting Tm (°C)
β-actin	Forward 5′-CAATAGTGATGACCTGGCCGT-3′ Reverse 5′-AGAGGGAAATCGTGCGTGAC-3′	60
NLRP1B	Forward 5′-AGGCAAGCACATTTGACTTCAAGGT-3′ Reverse 5′-GCTCAGTAGCTGCATCTTGCATTTC-3′	60
NLRC4	Forward 5′-GCGGAGGTGGGAGATATG-3′ Reverse 5′-CGTAGAAGGTTTGGAACAGC-3′	60
Caspase-1	Forward 5′-GGACTCTCAGCAGCTCCTCAGGCA-3′ Reverse 5′-GCAAAGCTTGACATTCCCTTCTGAGCC-3′	60
IL-1β	Forward 5′-GACCTTCCAGGATGAGGACA-3′ Reverse 52032-AGGCCACAGGTATTTTGTCG-3′	60
IL-18	Forward 5′-ACAGTGAAGTAAGAGGACTGGCTG-3′ Reverse 5′-CAGGTGCATCCATTTCCTCAAAGG-3′	60
GSDMD	Forward 5′-CCAGTGCCTCCATGATGAATGTGT-3′ Reverse 5′-TCACCACAAACAGGTCATCCC-3′	60
